# Physiology and Pathophysiology of Oxygen Sensitivity

**DOI:** 10.3390/antiox10071114

**Published:** 2021-07-12

**Authors:** Robert S. Fitzgerald, Asuncion Rocher

**Affiliations:** 1 Department of Environmental Health & Engineering, The Johns Hopkins University Medical Institutions, Baltimore, MD 21205, USA; 2 Departamento de Bioquimica y Biología Molecular y Fisiologia, Instituto de Biologia y Genetica Molecular (IBGM), Universidad de Valladolid-CSIC, 47005 Valladolid, Spain; rocher@ibgm.uva.es

**Keywords:** oxygen sensing, cardiovascular disease, carotid body, aortic bodies, astrocytes, hypoxia

## Abstract

Oxygen is an essential requirement for metabolism in mammals and many other animals. Therefore, pathways that sense a reduction in available oxygen are critical for organism survival. Higher mammals developed specialized organs to detect and respond to changes in O_2_ content to maintain gas homeostasis by balancing oxygen demand and supply. Here, we summarize the various oxygen sensors that have been identified in mammals (carotid body, aortic bodies, and astrocytes), by what mechanisms they detect oxygen and the cellular and molecular aspects of their function on control of respiratory and circulatory O_2_ transport that contribute to maintaining normal physiology. Finally, we discuss how dysregulation of oxygen availability leads to elevated signalling sensitivity in these systems and may contribute to the pathogenesis of chronic cardiovascular and respiratory diseases and many other disorders. Hence, too little oxygen, too much oxygen, and a malfunctioning sensitivity of receptors/sensors can create major pathophysiological problems for the organism.

## Identification of the Various Sensors of O_2_ in the Organism and by What Mechanisms They Sense O_2_: The Carotid Body, Aortic Bodies, and Astrocytes

The carotid body (CB) in humans is generally a tiny ovoidal structure with a diameter of only a few millimeters and weighing around 15 mg. It is referred to as aperipheral arterial chemoreceptor. This highly vascularized structure is located bilaterally near the bifurcation of the common carotid artery into its internal and external branches. Blood flow through the CB from a small branch off the external carotid artery is very high; in the cat it has been measured at >2 L/min/100 g tissue. Flow seems to exit into the internal jugular vein. In addition to the extensive vasculature the CB is composed of two cell types: the Type I or Glomus Cell (GC) and the Type II or Sustentacular Cell. The first is the primary chemo-detecting cell with vesicles containing neurotransmitters (e.g., acetylcholine [ACh], ATP, dopamine). Abutting the GCs are afferent neurons (dendrites in classical terminology), these neurons have cell bodies found in the petrosal ganglion. The axons from the cell bodies lead to the nucleus of the solitary tract in the medulla. From here, neural activity would be directed to vagal nuclei or sympathetic nervous system nuclei (e.g., paramedian reticular nucleus) in the medulla. A more recent investigation has found that the Type II cell not only supports but also participates in chemo-detection in a paracrine role.

Under normal, physiological conditions the CB is thought to sense low partial pressures of O_2_ in arterial blood (PaO_2_; hypoxia) by attenuating conductance in the K^+^ channels of the GCs. This retaining of positively charged ions depolarizes the GCs. When the membrane potential reaches a less negative value, voltage-gated calcium channels are opened and extracellular calcium rushes into the GCs and adheres to vesicles containing various neurotransmitters (e.g., ACh, ATP, dopamine). These vesicles proceed over to the inner surface of the GCs and attach thereon with the help of some form of protein (e.g., Synaptin1). The vesicles then exocytose their content into the synaptic cleft between the GC and an abutting neuron of the carotid sinus nerve, a branch of the 9th cranial nerve. The excitatory neurotransmitters, ACh and ATP, adhere postsynaptically to the appropriate cholinergic, or purinergic receptors and initiate an action potential which proceeds central.

Pathophysiological performance of the CB’s O_2_ sensitivity could develop in multiple loci in the above description of the normal or physiological pattern. But a very unique source of pathological performance was reported from Professor Harold Schultz’s lab at the University of Nebraska Medical Center in Omaha. It stemmed from a sensitization of the CB in chronic heart failure (CHF). In brief, they uncovered a mechanical sensor on the luminal surface of endothelial cells in the vasculature of the CB; the sensor detects the shear created by the flow of blood over the endothelial cell. The response to this by way of a cascade of proteins was to generate an increase in Kuppel Like Factor 2 in the CB. 

The aortic bodies (ABs) are tiny structures sprayed across the arch of the aorta. They are putative arterial chemoreceptors, and so do much the same as the CBs, but using different tools and organization. A recent study in the rat by Drs. Piskuric, Vollmer, and Nurse (McMaster University), has found glomus-like cells in whole mounts of juvenile rat vagus and recurrent laryngeal nerves (V-RL) and in dissociated cell culture. Glomus cells were routinely identified within these nerves. Many neuronal cell bodies and processes were closely associated with AB glomus clusters, especially near the V-RL bifurcation. Some neuronal cell bodies showed P2X2 and P2X3 purinoceptor subunits. These were also found in nerve terminals surrounding glomus cells. Glomus cells and apposed nerve terminals also detected in local neurons was a signal for the vesicular acetylcholine transporter (VAChT). Few neurons showed a positive signal for tyrosine hydroxylase or nNOS. Dissociated monolayer cultures of juvenile rat V-RL nerves yielded TH-immunoreactive glomus clusters with other processes and Type II cells showing glial fibrillary acidic protein. Cocultures survived for several days and expressed voltage-activated ionic currents and generated action potentials. Where the ABs respond to decreases in PaO_2_, they also respond to other forms of lowered O_2_ content such as one might find in a preparation exposed to carbon monoxide (CO). Hemoglobin (Hgb) has a much higher affinity for CO than for oxygen. So Hgb binds CO more tightly to loci ordinarily occupied by O_2_. CBs do not respond to a CO challenge, which is one explanation suggested for this difference between the CB and AB is blood flow. This is very rapid in the CB, but thought to be less so in the AB, though it is not clear that blood flow in the AB has ever been measured as it would be a very difficult measurement to make; metabolism in both sensors is high, but flow through the CB is so fast that apparently it can gather enough of its needed O_2_ from the dissolved fraction in the plasma.

Astrocytes, the most numerous of glial cells in the brain, respond rapidly to decreases in PO_2_ a few millimeters of mercury below normal brain oxygenation with elevations in intracellular calcium. This decrease inhibits astroglial mitochondrial respiration, leading to mitochondrial depolarization as well as several other effects, including the release of Ca^2+^ from intracellular stores; this last effect triggers fusion of vesicular compartments containing ATP. Blockade of astrocyte signaling by over-expression of ATP-degrading enzymes within the brainstem respiratory rhythm-generating circuits reveals the fundamental physiological role of astroglial oxygen sensitivity. In low oxygen conditions (excessive neuronal activity or a hypoxic environment) this mechanism increases breathing activity, even in the absence of peripheral chemoreceptor oxygen sensing.

What can be said regarding oxygen sensitivity of the central nervous system (CNS)? Certainly, all cells are sensitive to oxygen, simply to generate ATP needed to carry on their functions in that universe of neurons. If the CNS is deprived of oxygen, the individual becomes unconscious and faints. On the other hand, excess oxygen can also have dire consequences, such as oxygen toxicity. Oxygen toxicity is a condition resulting from the harmful effects of breathing oxygen sometimes at increased partial pressures. Severe cases can result in cell damage and death, with effects most often seen in the central nervous system and lungs. Historically, the central nervous system condition was called the Paul Bert effect, after the researcher who pioneered the discoveries and descriptions in the late 19th century.

The pulmonary apparatus is also a system very sensitive to oxygen levels. Under normoxic conditions the system functions quite freely without major problems, but when a gas containing, for example, only 15% oxygen is provided to the lung, one observes the classic pulmonary vasoconstrictor response. Alveolar hypoxia will elevate the normoxic pulmonary arterial pressure from about 15 mmHg to as much as 25 mmHg. However, it has long been known that this increase can be attenuated by a stimulus to the carotid body, a neuroreceptor located bilaterally near the bifurcation of the common carotid artery into its internal and external branches [1,2]. Some recent work has pointed out how the vascular resistance of the pulmonary vasculature can be influenced by the other set of arterial chemoreceptors, the aortic bodies (ABs), tiny structures sprayed across the arch of the aorta. During a hypoxic challenge to the organism, either from lowered P_a_O_2_ or from carbon monoxide, AB neural output increases. Removing the neural output from the ABs to the CNS produces an increase in pulmonary vascular resistance [3,4].

Cardiac tissue is sensitive to oxygen, as are vascular tissues (arterioles). Without oxygen these structures relax and are unable to respond to any input from a systemic reflex response; the reason is they are without ATP, ordinarily generated via the mitochondrial electron transport chain having oxygen in the final steps. In a word, without oxygen the oxidation of glucose, fatty acids or ketone bodies cannot happen. And cells structural and functional protein units would also fail to maintain a homeostatic, normal internal environment.

In congestive heart failure (CHF), blood flow throughout the organism is less than normal; the heart is, to variable degrees, unable to pump a normal cardiac output. This condition can sensitize the key receptor of the organism monitoring its internal environment, the CBs. These structures are located bilaterally at the juncture of the common carotid artery’s bifurcation into its external and internal branches. The CBs are often confused with the carotid sinuses. The carotid sinus, also bilaterally located, is a small expansion at the base of the of the internal carotid artery. The sinuses are the principal detectors and regulators of arterial blood pressure, whereas the CBs “taste” arterial blood for oxygen, carbon dioxide, hydrogen ion, and glucose. Clinical studies showing the results of life expectancy have reported in one group of CHF patients having sensitized CBs to be significantly less than a second group of CHF patients whose CBs were not sensitized. Sensitized CBs create a variety of abnormal conditions, e.g., irregular breathing patterns, sometimes resembling Cheyne–Stokes, unstable cardiac performance, malfunctioning kidneys. Highly innovative animal studies from the University of Nebraska Medical Center teased out the mechanisms behind the sensitization process [5]. Among their keen findings was the unique observation of what turned out to be a mechanical receptor found embedded in the luminal surface of endothelial cells in the CB’s vasculature. This sensor measures shear stress created by the flow of blood across it. This stimulus to the receptor activates a cascade of proteins, eventually activating Kruppel Like Factor 2 (KLF2). KLF2 proceeds more deeply into the cell to activate a NOS molecule generating NO. This molecule has been shown to attenuate the increase in neural output from the CB under a hypoxic challenge and this effect appears to stem, at least in part, from NO’s ability to reduce the release of ACh and ATP, excitatory neurotransmitters stored in vesicles in the CB’s glomus cells. During a hypoxic challenge, ACh and ATP are exocytosed into the synaptic cleft between GCs and the sensory afferent neuron abutting the GC.

**Figure 1:**
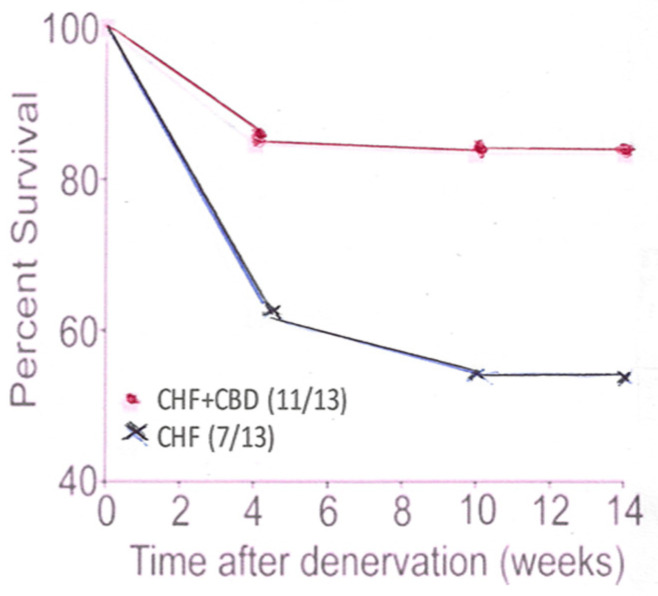
This panel shows the percent survival in CHF animals which had undergone CB denervation (CBD), compared to CHF animals which had not had their CB denervated.

Under conditions of CHF, blood flow throughout the organism, including the CB, is reduced in speed. Shear stress on the mechanical receptor is greatly reduced, or even lost. No KLF2 is generated and no NOS and, therefore, NO is lost with its attenuating effect. Procedures have been developed to confront this problem: (1) CB denervation by freezing it in situ (Figure 1); (2) administration of statins; (3) exercising the animals which increases the cardiac output and flow across the mechanical sensor on the luminal surface of the endothelial cells.

Carotid body influence over the cardiopulmonary system has several chapters frequently found in the literature. Less frequent but very important is the CB influence on sodium metabolism. Professor Honig has produced over several decades of study a very impressive corpus of literature treating CBs and natriuresis [6]. For example, CBs exert some influence over sodium metabolism, even in healthy individuals at sea level, the so-called “resting drive”. Inactivation of CBs of the cat is followed by a significant decrease in sodium excretion.

Honig also thought from work in cats that when the chemosensory resting drive is abolished, salt and water are retained. If this happens in humans, CBs could be involved in the aetiology of systemic hypertension. Honig also suggested that any reduction in the activity of the CBs could lead to an enhanced voluntary sodium intake, as well as a reduced ability of the kidney to excrete sodium and water. This increase in plasma volume could lead to primary systemic hypertension.

Hence, too little oxygen, too much oxygen, and a malfunctioning sensitivity of receptors/sensors can create major pathophysiological problems for the organism.

## Data Availability

The data is contained within the article.
